# The assessment of the effect of different intraabdominal pressures used for laparoscopic cholecystectomy surgery on optic nerve sheath diameter: a prospective observational cohort study

**DOI:** 10.3906/sag-2009-2

**Published:** 2021-06-28

**Authors:** Tuna ERTÜRK, Bülent Barış GÜVEN, Yadigar YILMAZ, Fulya YURTSEVER, Ayşın ERSOY

**Affiliations:** 1 Department of Anesthesiology, University of Health Sciences, Sultan 2. Abdulhamid Han Training and Research Hospital, İstanbul Turkey

**Keywords:** Optical nerve sheath diameter, intracranial pressure, laparoscopic cholecystectomy, ultrasonography

## Abstract

**Background/aim:**

During laparoscopic cholecystectomy operations, increases in intraabdominal, intrathoracic, and intracranial pressures (ICP) can be seen after pneumoperitoneum created for surgical imaging. Orbital ultrasonography (USG), which has been developed in recent years, is a method that can evaluate the ICP by measuring the optic nerve sheath diameter (ONSD) from the eyeball.In our study, we aimed to evaluate whether different intraabdominal pressure values created during laparoscopic cholecystectomy operations correlate with ICP by measuring ONSD.

**Materials and methods:**

The study included a total of 90 patients with American Society of Anesthesiologists (ASA) physical status classification I (ASA I) and II (ASA II) and ages from 18 to 65 years with laparoscopic cholecystectomy planned.After the patients were intubated, at the 5th min, bilateral ONSD measurements were performed. The same measurements were performed at the 15th and 30th min after CO2 insufflation and additionally 10 min after CO2 was released at the end of the operation. During intrabdominal CO2 insufflation, patients with 10 mmHg pressure applied comprised Group 1, patients with 12 mmHg pressure applied comprised Group 2, and patients with 14 mmHg pressure applied comprised Group 3.

**Results:**

The study was completed with 89 patients, 51 female and 38 males. One patient was excluded from the study due to erroneous values. The variations in ONSD measured in the right-left eye before pneumoperitoneum and at the 15th and 30th min after abdominal CO2 insufflation were observed to be statistically significant (p < 0.01). In all three groups, the right and left eye ONSD values were not identified to be statistically significantly different (p > 0.01).A significant increase was observed in ONSD values in direct proportion to the increase in intraabdominal pressure in patients undergoing laparoscopic cholecystectomy surgery.

**Conclusion:**

USG-guided ONSD measurements appear be a guide to ensure optimization of intraabdominal pressures and safe anesthesia administration for patients, especially those at risk of ICP increase, during laparoscopic surgery.

## 1. Introduction

With the introduction of laparoscopic surgery, laparoscopic cholecystectomy has entered practice as the gold standard for gallstone and gallbladder diseases [1]. Laparoscopic cholecystectomy is a minimally invasive surgical procedure to remove the gallbladder and became the preferred technique over open cholecystectomy at the beginning of the 1990s [2]. The laparoscopic cholecystectomy method is used for indications like cholecystitis (acute/chronic), symptomatic cholelithiasis, bile dyskinesia, acalculous cholecystitis, gallstone pancreatitis, and gallbladder masses or polyp treatment [3].

During laparoscopy, a range of respiratory, hemodynamic, and metabolic changes occur linked to pneumoperitoneum and patient position. These changes make anesthesia administration complicated [4]. After CO2 insufflation, increases may be observed in intraabdominal, intrathoracic, and intracranial pressures (ICP). Although most of these can be monitored with advanced monitoring techniques, these monitoring systems are insufficient to observe changes in the intracranial cavity.

To measure ICP, many techniques like transcranial Doppler, B-mode transcranial sonography, and orbital sonography have been developed. Orbital ultrasonography (USG) is a method developed in recent years that can evaluate ICP with optic nerve sheath diameter (ONSD) measurement through the eyeball which requires support by studies. Ultrasonographic measurement of ONSD is a noninvasive, simple, and rapid way to detect pressure changes of the intracranial compartment. A series of studies have been accomplished comparing the ultrasonographic measurement of the ONSD with neuroimaging in adults [5–9]. In one study, the ONSD/ICP correlation was found to be 0.685 (95% confidence interval [CI]: 0.626–0.737) [10]. In a study in children, sixty-four patients were recruited, of whom 24 (37%) had a confirmed diagnosis of increased ICP. The sensitivity of ONSD as a screening test for increased ICP was 83% (95% CI: 0.60 to 0.94); specificity was 38% (95% CI: 0.23 to 0.54); positive likelihood ratio was 1.32 (95% CI: 0.97 to 1.79); and negative likelihood ratio was 0.46 (95% CI: 0.18 to 1.23) [11,12]. Most of these studies have considered an upper normal ONSD limit of 5 mm. This cut-off point was proposed for patients above age 4 by the first validation studies on the ONSD and was found to have a good accuracy as a predictor of intracranial hypertension, with a sensitivity ranging from 70.8% to 100% and a specificity ranging from 63% to 100% [13,14]. 

In our study, we aimed to evaluate whether ICP was directly proportional to an increase in intraabdominal pressure created by CO2 pneumoperitoneum with 10 mmHg, 12 mmHg, and 14 mmHg pressures during primary laparoscopic cholecystectomy operations. The secondary purpose of the study is to evaluate the ICP changes that may occur due to increased abdominal pressure with USG-assisted ONSD measurement and to determine the most appropriate abdominal pressure to be applied for pneumoperitoneum in patients with increased ICP.

## 2. Materials and methods

This single-center prospective observational clinical study included 90 patients with laparoscopic cholecystectomy surgery planned with general anesthesia under elective conditions in American Society of Anesthesiologists (ASA) physical status classification I (ASA I) and II (ASA II), aged from 18 to 65 years. Written informed consent was obtained from each patient.

Patients with ASA III and ASA IV, previous abdominal-orbital trauma or surgical history, glaucoma, without consent given by themselves or legal heirs, known intracranial space-covering lesion, with high ICP, and with emergency surgery planned were not included in the study.

Preoperative evaluation noted name-surname, age, sex, date of operation, height-weight values, and ASA classification before entry into the operating room for all patients with no solid or liquid food intake for at least 6 h. Later, patients on the operating table had standard monitoring (electrocardiogram, noninvasive arterial blood pressure, peripheral oxygen saturation, and bispectral index (BIS)) followed by anesthesia induction (intravenous propofol 2 mg kg–1, rocuronium 0.5 mg kg–1, fentanyl 1 µg kg–1). After sufficient muscle relaxation was present, patients were intubated by the anesthesiologist and maintenance anesthesia with titration of sevoflurane, 50% O2–50% air, and remifentanil infusion of 0.05–0.1 µg kg–1 min–1 was planned to keep BIS values between 40 and 60.

At the 5th min after intubation, bilateral ONSD was measured before intraabdominal CO2 insufflation was completed by the surgical team with a Veress needle and the intraumbilical method. These measurements were recorded as basal values. Mechanical ventilator settings were tidal volume 8 mL kg–1, frequency 13, positive end expiratory pressure (PEEP) 5 in the volume control mode and 50% oxygen–50% air mixture was used. End tidal carbon dioxide (Et-CO2) was held in the interval of 30–40 mmHg. Airway peak and plateau pressure values were recorded. The same measurements were made at the 15th and 30th min after CO2 insufflation and 10 min after CO2 was released at the end of the operation. During intrabdominal CO2 insufflation, patients with 10 mmHg pressure were included in Group 1, patients with 12 mmHg pressure were included in Group 2, and patients with 14 mmHg pressure were included in Group 3. Single and careful measurement is made for all ONSD values. 

ONSD measurement was performed using USG (SonoSite M-Turbo HFL50x/15-6 MHz Lineer Transducer SonoSite, Inc. Bothell, WA 98021 USA) with the same anesthesiologist (Dr Tuna ERTÜRK) responsible for the general surgery operating room who previously performed this measurement on 30 patients. At all-time points when ONSD was measured, heart rate (HR), mean arterial pressure (MAP), peripheral oxygen saturation (SpO2), end tidal carbon dioxide values (Et-CO2), respiration rate, and air way peak and plateau pressure values were recorded.

**Figure 1:**
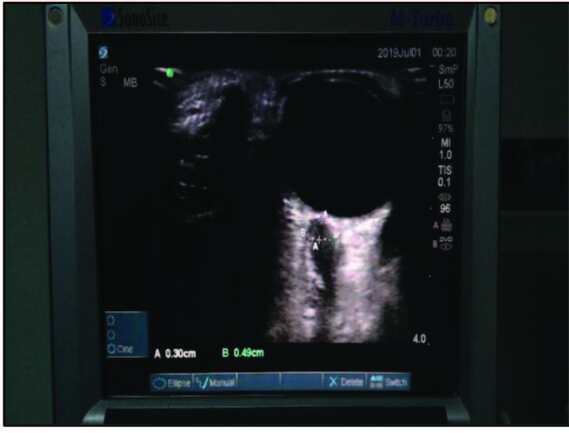
Ultrasonographic ONSD measurement.

**Figure 2:**
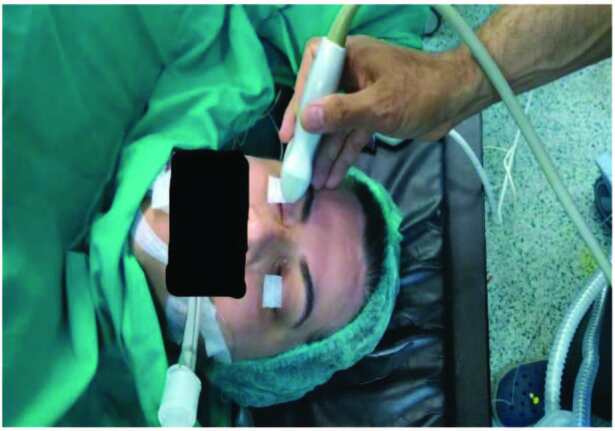
ONSD measurement, ultrasound position.

During ONSD measurement, after the patients were placed on the surgical table in supine position and intubated, they were given the 15° left and 20° reverse-Trendelenburg position in which the operation would be performed. Ultrasound gel was spread on the closed eyelids and a 15-6 MHz linear probe was placed on the temporal section of the eyelid. Bilateral measurements were performed by avoiding placing excess pressure on the eye (Figures 1 and 2). During measurement, the USG monitor was set to 4 cm depth and image in B mode. The appropriate angle was given to the probe to allow visualization of the eyeball and optic nerve on the monitor. On USG images, the ONSD was measured 3 mm below the junction site between the eyeball and optic nerve on a transverse line bilaterally, and values were recorded.

At the end of surgery, the patients were routinely extubated and taken to the postsurgery care unit. The modified Aldrete score (MAS) was used for patient recovery and patients with a score of 9–10 were transferred to the ward on trolleys.

### 2.1. Statistical analysis

After data were uploaded to a computer environment, analyses were performed with the SPSS v. 20.0 statistical program (IBM Corp., Armonk, NY, USA). When evaluating the data, descriptive statistical methods (frequency, percentage, mean, standard deviation) were used. Conformity to normal distribution was tested with the Kolmogorov–Smirnov and Kurtosis–Skewness coefficients. Then, continuous variables were compared with ANOVA and post hoc Tukey. For comparison of categoric variables, the chi-square test was used to determine differences between groups. The results were evaluated at the 95% confidence interval and the significance level at p < 0.05.

Based on our unpublished preliminary study average ONSD results, when calculated as α error: 0.05, power: 0.80, and effect size 0.40 according to the power analysis performed with the G * power package program, the sample size was 22 for each group and 66 in total.

## 3. Results

The study was completed with 89 patients, 51 female and 38 males. When all descriptive data are compared, there were no statistically significant differences found between the groups (p > 0.05) (Table 1).

**Table 1:** Comparison of descriptive data between the study groups.

	Group 1:P = 10 cmH2O(n = 30)	Group 2:P = 12 cmH2O(n = 29)	Group 3:P = 14 cmH2O(n = 30)	p value
Female; n (%)	15 (50%)	20 (69%)	16 (53.3%)	0.292a
ASA-I; n (%)	12 (40%)	12 (41.4%)	14 (46.7%)	0.859a
Age, years; mean ± SD	48.43 ± 13.00	51.27 ± 12.97	47.53 ± 12.61	0.511b
Height, cm; mean ± SD	169.10 ± 7.97	166.13 ± 8.19	168.56 ± 8.12	0.332b
Weight, kg; mean ± SD	75.40 ± 8.82	72.96 ± 11.62	77.26 ± 11.61	0.311b
BMI, kg/m2; mean ± SD	26.40 ± 3.04	26.42 ± 3.73	27.12 ± 3.20	0.640b

ASA-I: American Society of Anesthesiologists physical status classification-I, BMI: Body Mass Index, a: Chi-square test, b: One-way ANOVA test.

There were no statistically significant differences between the groups in terms of MAP, HR, P-peak, P-plateau, and etCO2 parameters (p > 0.05). However, there were statistically significant differences between the groups in terms of SpO2 saturation (p = 0.022) (Table 2).

**Table 2:** Comparison of hemodynamic data between the study groups before pneumoperitoneum.

	Group 1:P = 10 cmH2O	Group 2:P = 12 cmH2O	Group 3:P = 14 cmH2O	p value
Mean arterial pressure, mmHg; mean ± SD	82.93 ± 10.86	82.24 ± 10.75	86.46 ± 9.32	0.347a
Heart rate, beats/min; mean ± SD	71.43 ± 11.11	67.34 ± 12.04	69.76 ± 11.06	0.389a
Pressure-peak, cmH2O; mean ± SD	20.70 ± 1.62	21.41 ± 2.79	21.10 ± 2.39	0.498a
Pressure-plateau, cmH2O; mean ± SD	9.66 ± 0.71	10.10 ± 1.04	9.90 ± 0.99	0.200a
SpO2 saturation, %; mean ± SD	97.56 ± 1.19	97.13 ± 1.90	98.20 ± 1.15	0.022*, a, €
End-tidal CO2, %; mean ± SD	33.30 ± 1.82	32.51 ± 2.92	33.36 ± 2.18	0.310a

a: One-way ANOVA test, *: p < 0.05, €: Group II & Group III = p < 0.05.

There were statistically significant differences observed in variations in right-left eye ONSD measured before pneumoperitoneum and at the 15th and 30th min after pneumoperitoneum (p < 0.05) (Table 3).

**Table 3:** Comparison of ONSD Variation caused by pneumoperitoneum between the study groups.

	Group 1:P = 10 cmH2O	Group 2:P = 12 cmH2O	Group 3:P = 14 cmH2O	p value
Difference between ONSD measurement before pneumoperitoneum and at the 15th min of pneumoperitoneum				
Left eye, mm; mean ± SEM	0.050 ± 0.082	0.196 ± 0.018	0.376 ± 0.218	p < 0.001*, a, ¥, β, €
Right eye, mm; mean ± SEM	0.050 ± 0.177	0.134 ± 0.234	0.456 ± 0.274	p < 0.001*, a, ¥, β, €
Difference between ONSD measurements before pneumoperitoneum and at the 30th min of pneumoperitoneum				
Left eye, mm; mean ± SEM	0.053 ± 0.190	0.206 ± 0.237	0.463 ± 0.363	p < 0.001*, a, ¥, β, €
Right eye, mm; mean ± SEM	0.060 ± 0.282	0.165 ± 0.239	0.480 ± 0.304	p < 0.001*, a, ¥, β, €
Difference between ONSD measurements before pneumoperitoneum and after pneumoperitoneum				
Left eye, mm; mean ± SEM	0.003 ± 0.182	0.048 ± 0.241	0.010 ± 0.322	p: 0.342a
Right eye, mm; mean ± SEM	0.010 ± 0.193	0.020 ± 0.250	0.040 ± 0.347	p: 0.423a

ONSD: optic nerve sheath diameter, a: One-way ANOVA test, *: p < 0.05, ¥: Group I & Group II = p < 0.05,β: Group I & Group III = p < 0.05, €: Group II & Group III = p < 0.05.

In all three groups, there were no statistically significant differences identified for right and left ONSD values (p > 0.05) (Table 4).

**Table 4:** Comparison of right and left eye optic nerve sheath diameters of study groups before pneumoperitoneum.

	Group 1:P = 10 cmH2O	Group 2:P = 12 cmH2O	Group 3:P = 14 cmH2O	p value
Left eye ONSD, mm; mean ± SD	4.790 ± 0.347	4.572 ± 0.496	4.583 ± 0.494	0.117a
Right eye ONSD, mm; mean ± SD	4.796 ± 0.333	4.606 ± 0.502	4.540 ± 0.488	0.076a

ONSD: optic nerve sheath diameter, a: One-way ANOVA test.

## 4. Discussion

In the current study, we evaluated the effects of different intraabdominal pressures during pneumoperitoneum and ONSD measurements. We detected significant increases in ONSD at the increased abdominal pressures. As the applied pressure value increased, the increase in ONSD values became more pronounced.

Currently, laparoscopic interventions have begun to be chosen more often than conventional methods. However, practices like intraabdominal CO2 insufflation, ensuring imaging success, may involve a range of complications. Intraabdominal pressure increase occurring with CO2 insufflation increases intrathoracic and ICPs [15]. 

Studies support that ONSD can be a noninvasive imaging method that can evaluate ICP elevation timely [16–20]. Many studies have confirmed that ONSD changes with the changes in ICP [19–22]. Several studies have addressed specifically how ONSD is affected by acute changes in ICP. Findings showed that the change in ICP was reflected in ONSD measurement rapidly [16,23]. In our study, although there was no change in ONSD measurements taken at the 5th min after intubation, we observed statistically significant differences between the right-left eye ONSD changes measured at the 15th and 30th min after pneumoperitoneum was created. Secondary to this, we thought that the increase in intrabdominal pressure indirectly increased the ICP.

Monitoring of ICP changes is not included among standard monitoring methods for anesthesia. Noninvasive sonographic measurement of ONSD for determination of high ICP has high sensitivity-specificity values and is reported to be an appropriate, accurate, and noninvasive screening tool [10,11]. Orbital sonography is completed with a high-frequency linear transducer in B-mode and imaging of the nerve is made perpendicular to the eyeball. Diameter measurement should be performed 3 mm behind the optic disk [24]. It is a technique that is easy to learn and apply, with measurement of 25 patients generally sufficient for experience [25]. Before starting our study, we measured ONSD in 30 patients.

In the references, normal values of ONSD are given as 4 mm upper limit for under 1 year old, 4.5 mm for 1–14 years old, and 5 mm for older ages [11]. In our study, the mean ONSD value was 4.790 ± 0.347 mm for left eye and 4.796 ± 0.333 mm for the right eye in Group 1; 4.572 ± 0.496 mm for the left eye and 4.606 ± 0.502 mm for the right eye in Group 2; and 4.583 ± 0.494 mm for the left eye and 4.540 ± 0.488 mm for the right eye in Group 3. These values are somewhat below the values in the previous study.

Normally, there is interocular symmetry in both eyes for the ONSD; however, differences in ONSD are reported in situations with unilateral papillary edema or without any cause [26]. As a result, ONSD measurement with USG should be performed in both eyes. Therefore, in our study, we also measured ONSD in both eyes in each patient and we did not observe a statistically significant difference between the changes in right and left eye ONSD.

Although Haris et al. [27] reported increases in central venous pressure, mean arterial pressure and systemic vascular resistance linked to intraabdominal CO2 insufflation. In our study, there were no statistically significant differences identified between the hemodynamic data in the three groups.

Robba et al. [28] stated in their study that ICP would increase if the pneumoperitoneum and Trendelenburg position were applied together. In our study, during pneumoperitoneum with pressures at 10 mmHg, 12 mmHg, and 14 mmHg, the ONSD was directly proportional to the increase in abdominal pressure in spite of reverse Trendelenburg position; as a result, it was identified that intracranial blood pressure levels increased.

In their study with 61 patients, Sahay et al. [29] stated that there was an increase in ICP with pneumoperitoneum and Trendelenburg position and that it took at least 5 min for these values to return to basal levels. Similarly, in our study, it was observed that the increase in ONSD of the patients approached basal values as of the 10th min after evacuating the CO2 from the abdomen. 

The results of our study should be considered within some limitations. In the study, a direct measurement tool could be used in addition to an ICP indirect measurement tool or orbital sonographic measurement could be supported by the tonometer which is also an indirect measuring device. A larger sample could be taken into the study. One week after the surgery is over, another measurement could be taken, so that information about when ONSD returned to normal could be obtained. 

## 5. Conclusion

In conclusion, a significant increase was observed in ONSD values in direct proportion to the intrabdominal pressure increase in patients undergoing laparoscopic cholecystectomy surgery. As the applied pressure value increased, the increase in ONSD values became more pronounced.

Noninvasive ONSD measurements performed with USG can be a guide in terms of optimization of intraabdominal pressures, especially in laparoscopic surgeries (patients with head trauma, CVS, known-unknown intracranial pathology, etc.) where increased ICP poses a risk. In this way, safer anesthesia can be applied. Additionally, low pneumoperitoneum pressures should be chosen to preserve ICP in laparoscopic surgeries, especially in patients with high ICP. 

Ethical approval was obtained from the local ethical committee of University of Health Sciences Okmeydanı Training and Research Hospital, Clinical Research Ethics Committee (Ethics Committee document no: 48670771–514.10) and study was completed at University of Health Sciences Sultan 2. Abdülhamid Han Training and Research Hospital, Department of Anesthesiology.
